# S135. THE PSYCHOSIS CONTINUUM IN ELDERLY NON DEMENTED PERSONS: EVIDENCE DERIVED FROM PSYCHOTIC SYMPTOMS IN OLDER PEOPLE WITHOUT DEMENTIA FROM A BRAZILIAN COMMUNITY-BASED SAMPLE: A SEVEN YEARS’ FOLLOW-UP

**DOI:** 10.1093/schbul/sby018.922

**Published:** 2018-04-01

**Authors:** Walter Soares, Eriton dos Santos, Cassio Bottino, Helio Elkis

**Affiliations:** University of São Paulo

## Abstract

**Background:**

The idea of psychosis as a dichotomous entity is generally accepted, but general population studies samples have shown individuals reporting psychotic symptoms that do not fulfill clinical criteria for any disorder. Literature suggests that psychosis phenotype is almost 50 times higher than the dichotomous psychosis concept and are more frequent in persons with lower age, lower level of education or quality of life. The prevalence of hallucinations and delusions is higher than the prevalence of psychotic clinical disorders thus suggesting evidence of a psychosis continuum, which is not adequately appreciated in the literature, particularly in elderly populations.

The purpose of the present study was to evaluate a cohort of an elderly population during a seven year follow up study aiming to determine the incidence of psychotic symptoms and their correlations with somatic and cognitive clinical aspects.

**Methods:**

This is cohort study of a community-based sample of elderly subjects. Patients were evaluated by standard clinical interviews, clinical and cognitive status including the Mini-Mental State Evaluation (MMSE). At study entry in 2004, the sample was composed of 1,125 individuals aged 60 years and older. Of this total, 547 subjects were re-evaluated in 2011 and submitted to the original study protocol. Of these, 199 showed no psychotic symptoms at phase I, while 64 already had psychotic symptoms in 2004.

**Results:**

The incidence of at least one psychotic symptom in the 7 years period was 8.0% and 1.0%. Visual/tactile hallucinations were the most frequent (4.5%), followed by persecutory delusions (3.0%) and auditory hallucinations (2.5%). Individuals that reported persecutory delusions had the lowest MMSE mean score (19.00). Epilepsy was a predictive variable for auditory and visual/tactile hallucinations (OR: 7.75 and 15.83); lower MMSE (OR: 0.72) and reported depression (OR: 6.48) were predictive for persecutory delusions. Visual/tactile hallucinations were predictive of cognitive impairment conversion (OR: 5.66). A total of 57.8% of individuals with psychotic symptoms developed cognitive impairment after 7 years.

**Discussion:**

The presence (incidence) of subclinical psychotic symptoms in an elderly sample of a developing country like Brazil as well as the conversion rate to cognitive impairment were higher than reported in other developed countries. Visual/tactile hallucination had a crucial position in this context, was the most frequent symptom reported and was the only psychotic symptom which could predict cognitive impairment after a period of 7 years. A significant relationship was found between the incidence of psychotic symptoms and low MMSE scores, as well as clinical comorbidities such as epilepsy and reported depression. These findings provide evidence for the psychosis continuum hypothesis among elders and contribute to elucidate risk factors for these symptoms expression and its relation to cognitive impairment conversion.

This is part of a Ph.D. thesis and the correspondent article was published in PLoS One journal (doi: 10.1371/journal.pone.0178471).

